# Recyclable Polydimethylsiloxane Network Crosslinked by Dynamic Transesterification Reaction

**DOI:** 10.1038/s41598-017-11485-6

**Published:** 2017-09-19

**Authors:** Huan Zhang, Chao Cai, Wenxing Liu, Dongdong Li, Jiawei Zhang, Ning Zhao, Jian Xu

**Affiliations:** 1 Beijing National Laboratory for Molecular Sciences, CAS Research/Education Center for Excellence in Molecular Sciences, Laboratory of Polymer Physics and Chemistry, Institute of Chemistry, Chinese Academy of Sciences, Beijing, 100190 China; 2 University of Chinese Academy of Sciences, Beijing, 100049 China; 3 Division of Polymer and Composite Materials, Ningbo Institute of Material Technology and Engineering, Chinese Academy of Sciences, Ningbo, 315201 China

## Abstract

This article reports preparation of a crosslinked polydimethylsiloxane (PDMS) network via dynamic transesterification reaction between PDMS-diglycidyl ether and pripol 1017 with Zn(OAc)_2_ as the catalyst. The thermal dynamic nature of the network was investigated by the creep-recovery and stress-relaxation tests. The synthesized PDMS elastomer showed excellent solvent resistance even under high temperature, and could be reprocessed by hot pressing at 180 °C with the mechanical properties maintained after 10 cycles. Application of the PDMS elastomer in constructing micro-patterned stamps repeatedly has been demonstrated. The high plastic temperature and good solvent resistance distinguish the research from other reported thermoplastic PDMS elastomers and broaden the practical application areas.

## Introduction

In recent years, smart materials showing shape memory^1–10^, reprocessing/recycling^11–19^, self-healing^20–32^, high stimuli-responsivity^33–35^ and other excellent properties^36–38^ have been developed based on dynamic chemistry^39–41^. Among these materials, polymer networks with recycling capacity have attracted great interests because the traditional polymer networks crosslinked by covalent bonds are unrecyclable, and are generally treated by landfilling or burning after their lifetime. In terms of both environmental and economic reasons, it is important to endow polymer networks recyclability.

Recycling polymers can be achieved through noncovalent bonds and reversible dissociation of chemical reactions, however, the materials based on these interactions will lose their structural integrity under heating because of the insufficient crosslinks density^11–13^. On the contrary, associative exchangeable covalent bonds or reactions, such as imine bond^14^, transalkylation^15^, transcarbamoylation^16^ and transamination^17^, can form dynamic crosslinks to realize recycling. Different from polymers crosslinked by noncovalent bonds and reversible dissociation of chemical reactions, the dynamic property of the network based on associative exchangeable covalent interactions is shown by the topology changing while the number of crosslinks keeps constant both in inter- and intra-molecular polymer chains. Transesterification as an exchangeable reaction is an equilibrium process, where the exchange between ester and alcohol creates a new pair of ester and alcohol through the alkoxy moiety interchange^42^. The reaction will be accelerated via the addition of catalysts, like acid, base and inorganic salts. Based on the reversible transesterification reaction, Leibler and co-workers^18^ designed epoxy networks with malleability, reparability, recyclability and insolubility. Then Qi and co-workers^19^ specifically demonstrated the reprocessing and recycling ability under 180 °C in the same system.

Silicone elastomers, which are commonly thermosets, have been widely used in diverse fields such as aerospace, automobile, pharmaceutical and food service industry, due to their heat resistance, chemical stability and abrasion resistance etc. Silicone elastomers can resist high temperature up to 200 °C for over 10000 sequential hours and can even be used at 300 °C for a short period^43^. To endow with silicone elastomers recyclability is a challenge. Recently, thermoplastic silicone elastomers created by employing dynamic chemistry have been reported^44–49^. However, their plasticizing temperatures were relatively low and could not meet the requirement for practical utilization. In addition, in thermoplastic silicone elastomers based on H-bonding or triblock copolymers^50,51^, melting temperatures could reach above 170 °C, but they may not have good solvent resistance at high temperature.

Herein, crosslinked PDMS network was obtained from the reaction between PDMS-diglycidyl ether and pripol 1017 catalyzed by Zn(OAc)_2_. Owing to the reversible transesterification reaction, the as-prepared network had excellent recyclability at 180 °C and the mechanical properties could be maintained even after 10 cycles. Example of employing the PDMS elastomer prepared as reusable stamps has been demonstrated. The high plastic temperature will broaden the scope of applications of the thermoplastic silicone elastomers.

## Results and Discussions

### Preparation and structural characterization of crosslinked PDMS network

**Figure 1:**
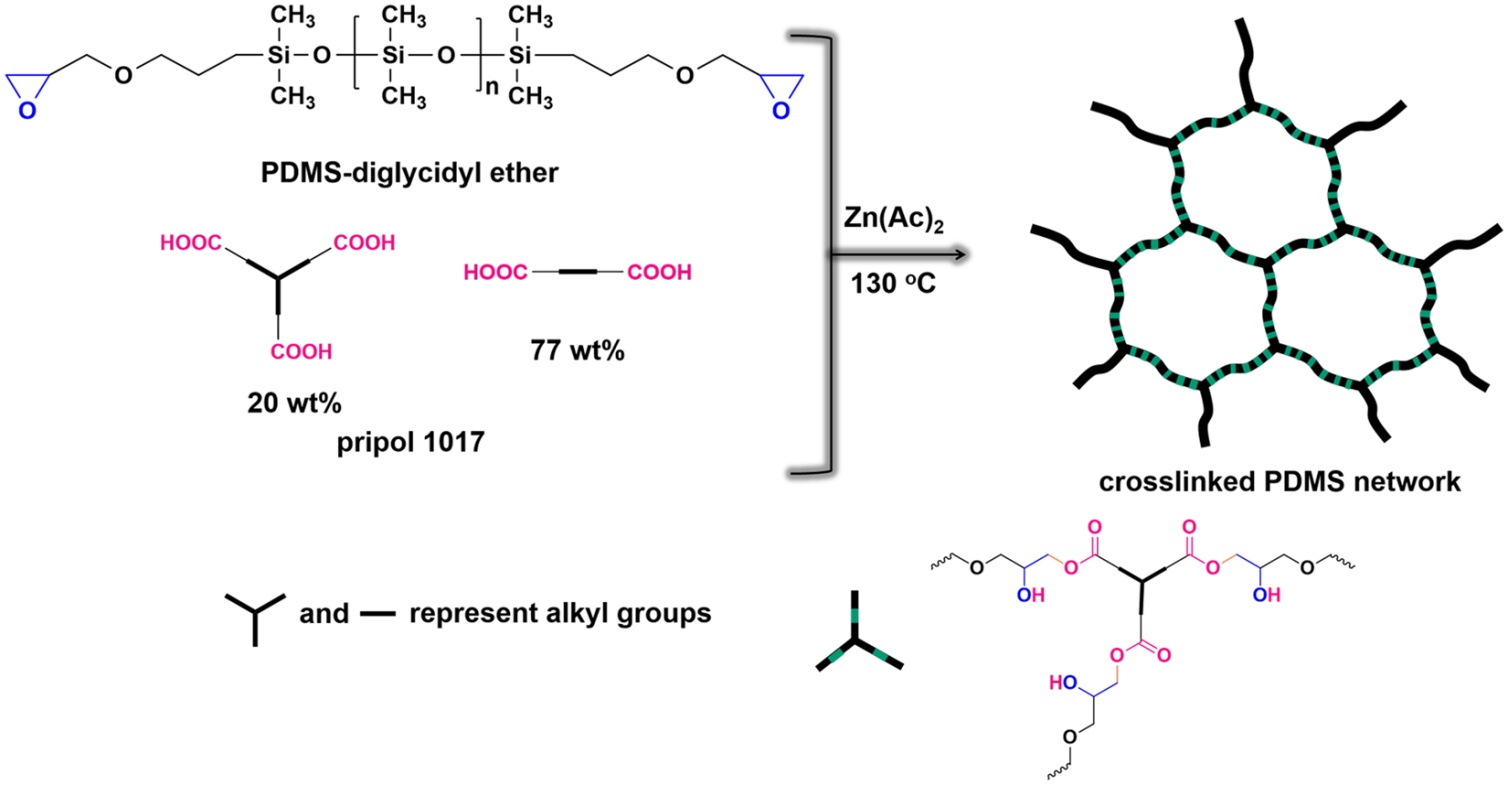
Synthesis of the crosslinked PDMS network.

The crosslinked PDMS network was prepared using the reaction between PDMS-diglycidyl ether and pripol 1017 with Zn(OAc)_2_ as the catalyst (Fig. 1). The crosslinking process completed after 12-hour at 130 °C, which was confirmed by the disappearance of typical epoxy ethyl peak (911 cm^−1^), the blue shift of acid peak (1710 cm^−1^) to ester peak (1740 cm^−1^) and the appearance of hydroxyl absorption peak at 3474 cm^−1^ in FT-IR spectra (see Supplementary Fig. S1), and the increasing proton peaks of epoxy ethyl from 2.5–3.2 to 3.9–4.3 ppm in ^1^H NMR results (see Supplementary Fig. S2).

### Swelling properties of crosslinked PDMS network

**Figure 2:**
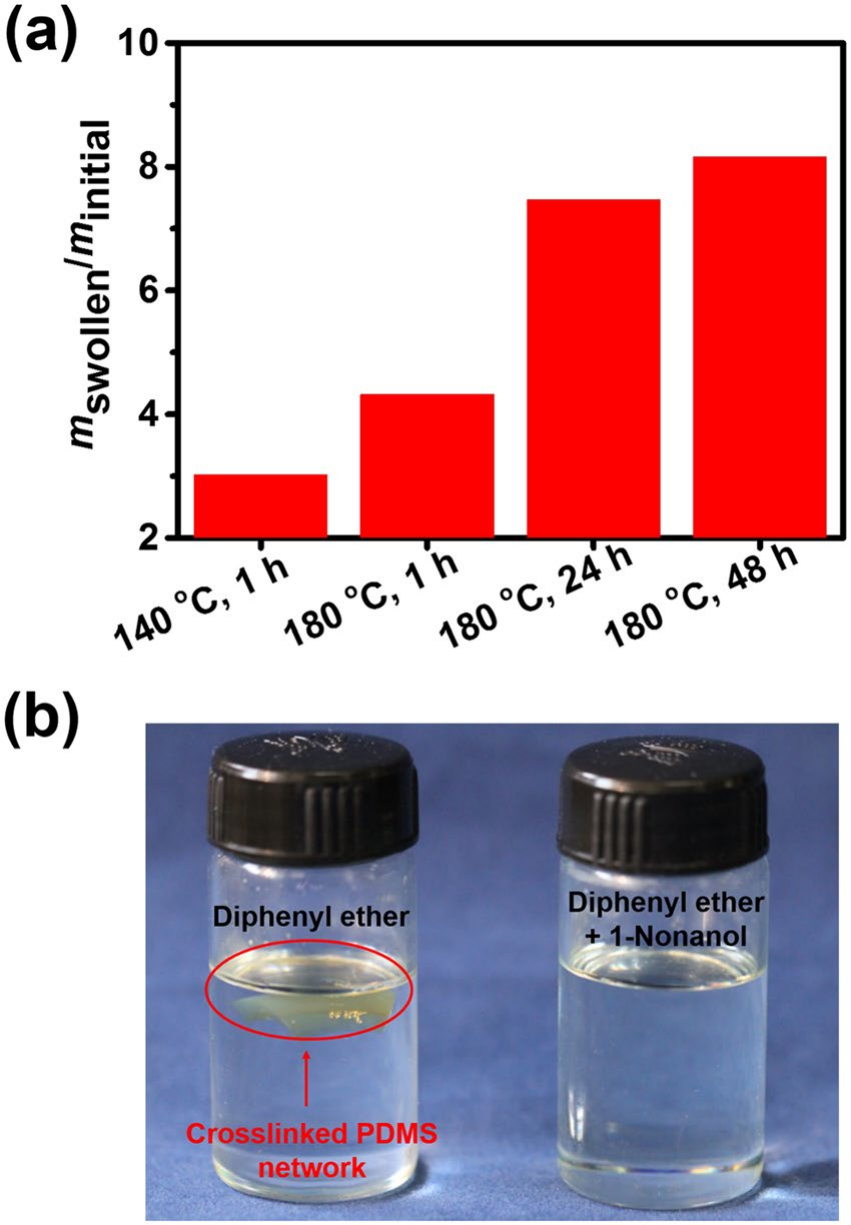
Solvent resistance of the crosslinked PDMS network. (**a**) Comparison of *m*_swollen_/*m*_initial_ of crosslinked PDMS network at different swelling temperatures and periods in diphenyl ether. (**b**) Digital photograph exhibiting the solubility using diphenyl ether (left) and diphenyl ether/1-nonanol (right) as the solvent at 180 °C.

When the obtained crosslinked PDMS network was swollen in its good solvent toluene, a swelling ratio (*SR*) of 355%, a gel fraction (*GF*) of 90% and a crosslink density (*ν*) of 0.0164 mol/cm^3^ were obtained (see Supplementary Table S1), indicating the integrity with few defects of the crosslinking structure. To determine the effects of crosslinking and de-crosslinking, solubility experiments in diphenyl ether were conducted at high temperature. The mass ratio of the crosslinked PDMS network after swelling (*m*_swollen_/*m*_initial_) increased with time (Fig. 2a). The crosslinked PDMS is insoluble at 180 °C for 48 h (Fig. 2b, left). This good solvent resistance at high temperature can be ascribed to that the transesterification reaction is a kind of associative exchangeable covalent interactions. Thus the number of crosslinks keeps constant both in inter- and intra-molecular polymer chains, making the network integrity in the solvent at 180°C. Nevertheless, a complete dissolution occurred by adding 1-nonanol into the solvent, due to the collapse of the network by the induced transesterification reaction between the network and the small molecules (Fig. 2b, right).

### Mechanical and thermal properties of crosslinked PDMS network

The elastic network at room temperature was yielded through the reaction of PDMS-diglycidyl ether and pripol 1017, leading to a glass transition temperature of about −50 °C (see Supplementary Fig. S3b), a storage modulus and a rubbery plateau of approximately 600 MPa and 0.71 MPa, respectively, (see Supplementary Fig. S3a), and a maximal stress of about 0.45 MPa (see Supplementary Fig. S9). The prepared network is thermally stable up to 250 °C with a weight loss of around 1.5% (see Supplementary Fig. S4a). Isothermal TGA was performed in case the crosslinked PDMS network might be used at elevated temperatures for a long-term (see Supplementary Fig. S4b). The weight loss at 130, 180 and 200 °C after 60 min is less than 1, 3 and 5%, respectively, demonstrating that the crosslinked PDMS network is thermally stable under the reasonable temperature for certain period.

### Dynamic nature of crosslinked PDMS network

**Figure 3:**
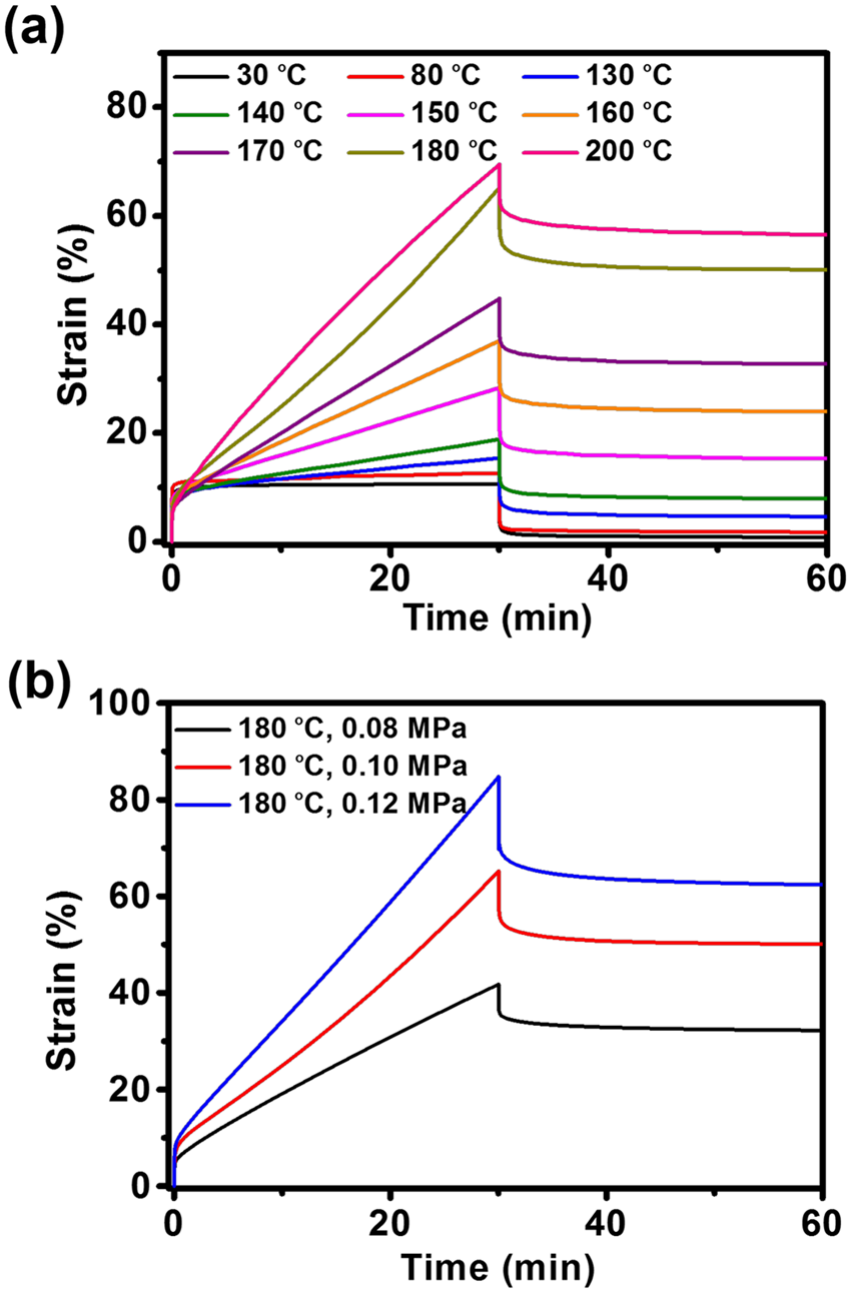
Creep-recovery curves (**a**) with an applied stress of 0.10 MPa at different temperatures and (**b**) at 180 °C with different stress levels for the crosslinked PDMS network.

The creep-recovery behaviors of the crosslinked PDMS network were examined to demonstrate the elastomeric performance. As shown in Fig. 3a, when a constant stress of 0.10 MPa was applied for 30 min, there is no noticeable creep for the temperature below 80 °C. The sample could recover its original dimension with a small residual strain (<15%, see Supplementary Fig. S5a) when it was recovered for 30 min after releasing the applied stress. The creep-recovery behaviors at higher temperatures are quite different under the same conditions. A slight level of creep is observed at 130 and 140 °C, further increasing temperature (150–200 °C) leads to an enhancement of creep levels and the slope of deformation strain. Meanwhile the second creep region where the creep strain is proportional to time can be observed clearly, indicating a typical viscoelastic liquid behavior of the crosslinked PDMS network. The phenomena might be ascribed to the slippage of the polymer chains in the network due to the triggering of the transesterification reaction at elevated temperatures. On account of the smaller degree of slippage of polymer chains at 130 and 140 °C, relatively higher recovery of deformation of 69% and 58% were observed, respectively. Yet, the dramatical decrease of strain-recovery ratio can be attributed to transesterification reaction when activated in the temperature range of 150–200 °C (see Supplementary Fig. S5a), consistent with the increased thermal energy initiating the rearrangement of ester bonds. Additionally, constant stress level is a key factor influencing the creep-recovery behaviors of viscoelastic materials^52^. Three different stress levels, namely 0.08, 0.10 and 0.12 MPa, were investigated at 180 °C (Fig. 3b). The constant stress increased from 0.08 to 0.12 MPa resulting in the strain increased from 42 to 85%. Importantly, the crosslinked PDMS network shows the characteristics of liquid because of the second creep stage dominated. These remarked results are proved to be derived from transesterification reaction induce the slippage of polymer chains much easier and faster under higher level stress. Thus the sample cannot return to its original dimension with a high residual strain. Moreover, the creep compliance which is represented in proportion to response strain was calculated. The creep compliance increases with the stress level (see Supplementary Fig. S5b), also implying the initiated transesterification reaction contributes to the non-linear viscoelastic behavior for crosslinked PDMS network. Hence, the creep-recovery experiments demonstrate that transesterification reaction can be triggered at elevated temperatures, which enables the recyclability of the crosslinked PDMS elastomer.

**Figure 4:**
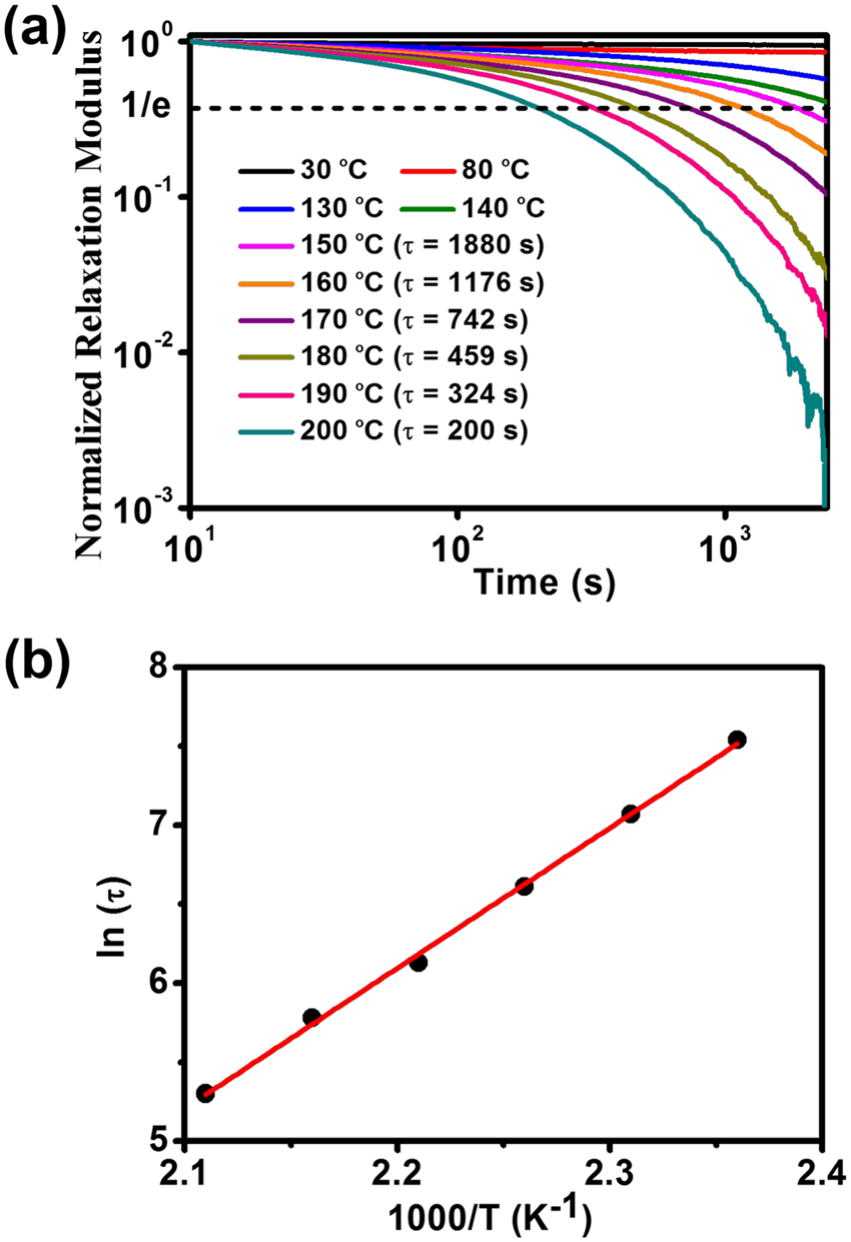
(**a**) Stress-relaxation curves of the crosslinked PDMS at different temperatures over a time period of 40 min. (**b**) Fitted Arrhenius equation (the red line) according to the experimental data (the black dots).

To further testify the reverisibility of transesterification reaction, stress-relaxation experiments were investigated in another manner. The samples were deformed to a certain strain (1%), and then the relaxation modulus at the temperature range of 30–200 °C was recorded (Fig. 4a). Obvious relaxations of stress are observed (130–200 °C), while the curves show almost flat profiles at lower temperature (30 and 80 °C). The slight relaxation observed at 130 and 140 °C is more likely due to the diffusion of dangling chains and a few slippage of polymer chains^53,54^. Whereas obvious stress relaxation is shown as the temperature further increased, which should be ascribed to transesterification reaction since the network structure and the degree of crosslinking maintain constant during the tests. Remarkably, the material performs an increasing relaxation rate with the increase of temperature. For instance, at 130 °C, the normalized relaxation modulus decreased from 1 to 0.63 MPa for 1800 s, however, during the same period the normalized relaxation modulus decreased by 99% due to the transesterification reaction initiated under 180 °C. The relaxation times were measured at normalized relaxation modulus of 37% (1/e) based on the Maxwell model for viscoelastic polymers^17^, which decreased from 1880 s at 150 °C to 200 s at 200 °C, due to the acceleration of transesterification reaction at higher temperature. Furthermore, the temperature dependence of the relaxation time is plotted by the Arrhenius equation^55^. As shown in Fig. 4b, the correlation between the relaxation time and temperature indeed fits the Arrhenius law, and the relevant activation energy (*E*_a_ ~ 74 kJ/mol) was calculated based on the slope.

### Thermal recyclability of crosslinked PDMS network

**Figure 5:**
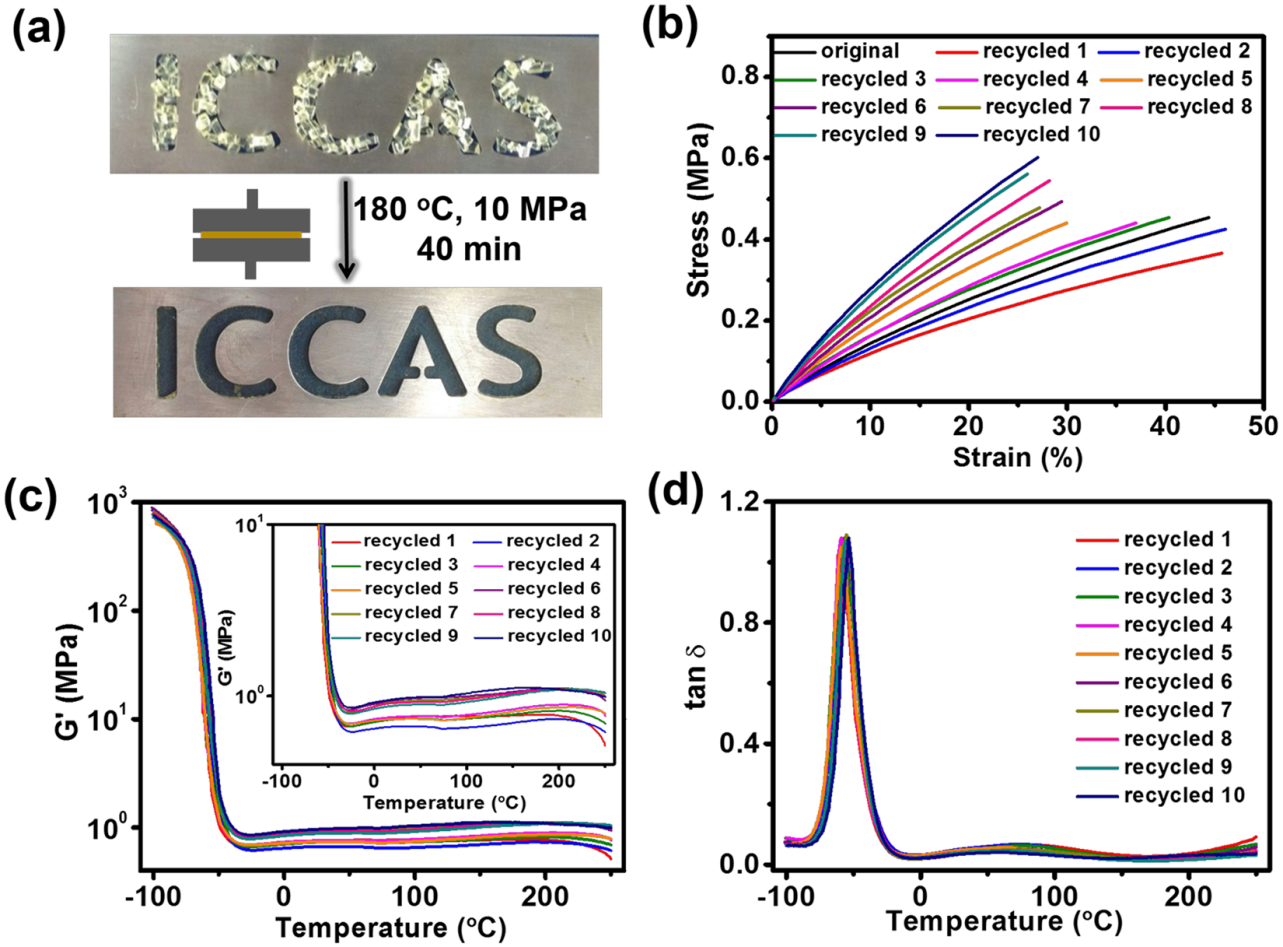
(**a**) Photo showing the thermal recycling of the crosslinked PDMS by hot pressing. (**b**) The stress-strain curves, temperature dependence of (**c**) storage modulus (G′) and (**d**) loss factor (tan *δ*) of the crosslinked PDMS network recorded in a 10-cycle recycling process.

The thermal dynamic nature of the transesterification reaction can facilitate the reprocessing of the crosslinked PDMS network. As shown in Fig. 5a, the sample was cut into small pieces, and then remolded to attain a defect-free appearance at 180 °C and 10 MPa for 40 min. Under the same conditions, a circular film could be remolded from a hexagon-shaped one (see Supplementary Fig. S6a,b), and the recycling process could be repeated for another nine times (see Supplementary Fig. S6c–k). Because of part aging of the crosslinked PDMS network at elevated temperature for a long period, the color of the sample turned slightly dark after several recycles. Furthermore, it is also noteworthy that annealing shows a profound influence in the properties of the recycled network. For example, the recycled PDMS film annealed at 130 °C for 12 h was stiffer than the one without thermal treatment (Fig. 5b, see Supplementary Fig. S7). This can be ascribed to the annealing helps the rearrangement of polymer network. To investigate the performances of the recycled materials, tensile tests, rheology measurements, swelling experiments and ATR-FT-IR were performed. Tensile tests shown in Fig. 5b indicate that the mechanical properties do not reduce obviously even after ten generations of recycling. However, the recycled polymer networks, which presumably underwent a structural rearrangement and partially aging at elevated temperature^56^, express enhancement in maximal stress and Young modulus (see Supplementary Fig. S9). The results of rheology measurements shown in Fig. 5c suggest the storage moduli have no significant change, but the rubbery plateaus display a slight increasing, which is in agreement with the tensile tests. The dependence of the loss tangents (tan *δ*) on the temperature of the recycled networks (Fig. 5d) indicates that the glass transition temperature is around −50 °C. The increasing recycling times resulted in the decrease of swelling ratio and the increase of gel fraction and crosslink density (see Supplementary Table S1). Thus, more integrated polymer networks with fewer defects were obtained due to the rearrangement of polymer network. ATR-FT-IR spectra did not change by recycles, showing that the recycled polymer networks have almost identical structures compared to the as-synthesize one (see Supplementary Fig. S8).

In comparison with previous publications^44–49^, the high thermoplastic temperature of the PDMS elastomer prepared shows obvious advantage when considered the heat resistance in practical application. For instance, Brook and co-workers^44^ demonstrated that thermoplastic silicone elastomers based on π-π stacking interactions could be remolded at 85 °C, and silicone boronates elastomers crosslinked by Lewis acid-Lewis base interaction could be reprocessed only at 60 °C^46^.

**Figure 6:**
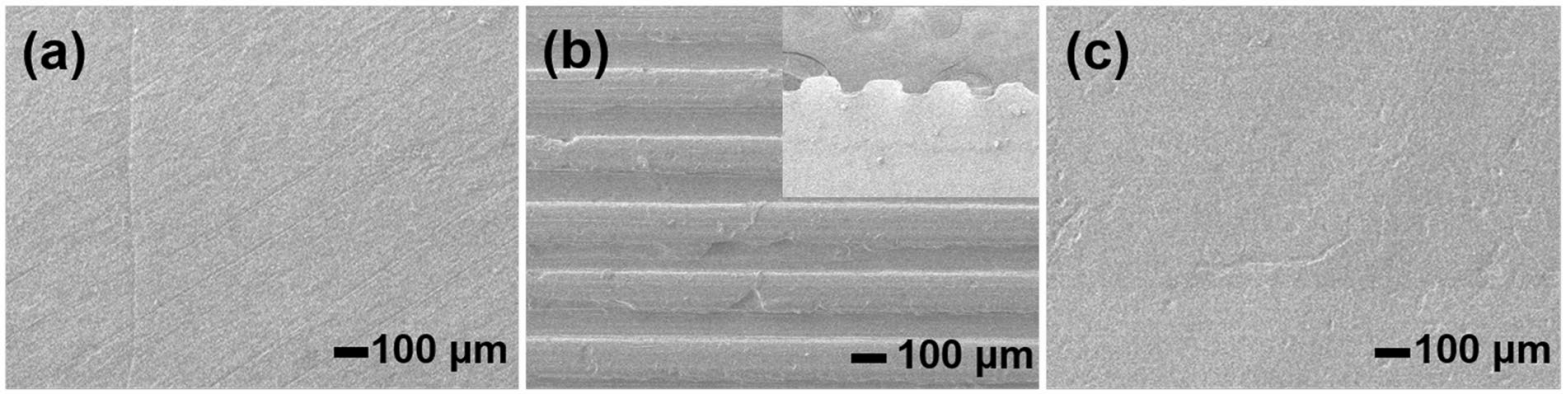
SEM images of (**a**) the original, (**b**) the patterned and (**c**) the recovered surfaces of the PDMS stamps.

PDMS is widely used to prepare elastic stamps with patterns on the surface^57,58^. However, stamps prepared by traditional PDMS lack the ability of reconstruction, thus recyclable PDMS stamp would be preferred from both economic and operational view^59^. Here, the PDMS elastomer prepared could be used repeatedly as a stamp material, as shown in Fig. 6. The initial PDMS stamp with a smooth surface (Fig. 6a) could be patterned to form periodical grooves by hot pressing at 180 °C using an aluminum template (Fig. 6b). The patterned PDMS stamp could be processed to a featureless morphology again by using a flat template under hot pressing (Fig. 6c).

## Conclusions

In conclusion, the thermally reversible crosslinked PDMS network was prepared via transesterification reaction between PDMS-diglycidyl ether and pripol 1017 catalyzed by Zn(OAc)_2_. The characteristics of the dynamic transesterification reaction endow the crosslinked network good solvent and heat resistances, compared to other reported thermoplastic PDMS elastomers^44–49^. Example of using the crosslinked PDMS as recyclable stamp material has been demonstrated. The introduction of transesterification reaction into PDMS network broadens the practical application areas of recycling silicone elastomers.

## Methods

Polydimethylsiloxane, diglycidyl ether terminated (PDMS-diglycidyl ether, *M*_n_ ~ 800, epoxide equivalent weight, 490) was purchased from Sigma-Aldrich. Pripol 1017 provided by Croda was a dimer fatty acid containing about 77 wt% dimers and 20 wt% trimers (acid value, 193 mgKOH/g). Zinc acetate dihydrate (Zn(OAc)_2_, 98%) was supplied by Strem Chemicals. 1-Nonanol (99%) was purchased from TCI. Diphenyl ether and toluene were provided by Beijing Chemical Works (China). All reagents were used without further purification.

In a 25 mL round-bottom flask at 100 °C, pripol 1017 (5.18 g, 17.6 mmol COOH group) and Zn(OAc)_2_ catalyst (0.19 g, 0.88 mmol, 5 mol% to COOH group) were added and stirred in vacuum. Temperature was gradually increased from 100 to 180 °C, and then was kept at 180 °C until no gas overflow could be observed and catalyst particles were fully dissolved (1–2 h). Then PDMS-diglycidyl ether (8.63 g, 17.6 mmol epoxy) was added and the temperature was kept at 130 °C under stirring until a homogeneous solution formed. The solution was quickly poured into Teflon dishes and kept at 130 °C for at least 12 h. A stretchy crosslinked PDMS film with the thickness of about 0.5 mm was obtained.

Fourier transform infrared (FT-IR) spectra were recorded on a Perkin-Elmer 2000 FT-IR spectrophotometer from 4000 to 400 cm^−1^ for 32 scans at a resolution of 2 cm^−1^. Samples of PDMS-diglycidyl ether and pripol 1017 were prepared by directly dropping the solution onto KBr films. Sample of crosslinked PDMS was obtained by dropping the mixture of PDMS-diglycidyl ether, pripol 1017 and catalyst onto KBr film, and then solidifying at 130 °C for 12 h. Attenuated total reflection fourier transform infrared (ATR-FT-IR) spectra of the crosslinked PDMS and the recycled ones were recorded in the range 600–4000 cm^−1^ at a resolution of 2 cm^−1^ for 32 scans.^1^H NMR investigation was carried out on a Bruker 400 M instrument with CDCl_3_ as the solvent at room temperature.

Thermogravimetric analysis (TGA) was performed using a PerkinElmer Pyris 1 TGA at a heating rate of 10 °C/min from 25 to 300 °C with an air flow rate of 20 mL/min. Isothermal TGA was performed at 130, 180 and 200 °C for 60 min. Scanning electron microscopy (SEM) images were obtained by using a JSM-7500F (JEOL, Japan) field-emission scanning electron microscope at an accelerating voltage of 5 kV.

Tensile tests were measured at 25 °C using rectangular-shape specimens with approximate dimensions of 25 mm × 4 mm × 0.5 mm in tensile mode on a Q800 DMA (TA Instrument). Stress/strain tests were conducted in DMA strain rate mode with a strain rate of 200%/min. For each measurement, at least three specimens were tested at the same condition.

Temperature sweep and stress relaxation experiments were performed on a Physical MCR 302 rheometer (Anton Paar, Austria) using an 8-mm parallel plate. In temperature tests, each sample was measured at an angular frequency of 1 rad/s and a constant strain amplitude in the linear viscoelastic region (−100–30 °C, *γ* = 0.05%; −30–250 °C, *γ* = 0.5%), using cooling rate of 3 °C/min. In stress relaxation tests, the samples were equilibrated for 5 min at a constant temperature, and then 1% strain amplitude in the linear viscoelastic region was applied and the stress was recorded over time. To ensure a good contact between materials and parallel plate, a constant normal force of 5 N was applied in the test.

Creep-recovery behavior was conducted using the tensile mode on Q800 DMA (TA Instrument). The rectangular-shape specimens (25 mm × 4 mm × 0.5 mm) were stretched for 30 min with an applied stress, and then the stress was released and the specimen would recover for another 30 min at a certain temperature.

Recycling test was carried out in a vacuum mould pressing machine (FM450, China). The original specimen was cut into small pieces and pressed into a 4 cm × 4 cm × 0.5 mm film under 10 MPa for 40 min at 180 °C, following by an annealing process at 130 °C for 12 h. The same procedure was repeated for another nine cycles.

Solubility experiments were conducted using diphenyl ether as the solvent at high temperature. The sample was immersed into diphenyl ether with the temperature kept at 140 and 180 °C for 1 h, respectively, then was kept soaking at 180 °C for another 47 h to reach the swelling equilibrium. Upon addition of 1-nonanol into the solvent, the sample could be dissolved completely in diphenyl ether at 180 °C.

Swelling experiments of the crosslinked PDMS network (*m*_initial_) were performed by immersing in toluene for 7 days to reach the swelling equilibrium (*m*_swollen_). The swelling process was under stirring and the solvent was replaced every 12 h. Subsequently, the swollen samples were dried at 50 °C in a vacuum oven until a constant weight *m*_dry_ was obtained. Hence, swelling ratio (*SR*) and gel fraction (*GF*) are calculated on the basis of the equations 1 and 2.

1 $$SR=\frac{{m}_{{\rm{swollen}}}-{m}_{{\rm{dry}}}}{{m}_{dry}}\times 100 \% $$

2 $$GF=\frac{{m}_{{\rm{dry}}}}{{m}_{{\rm{initial}}}}\times 100 \% \,$$

The equilibrium swelling experiment was used to determinate the crosslink density *ν* based on the Flory-Rehner Model^60–62^ (equation 3).

3 $$\nu =\frac{\mathrm{ln}(1-{\O }_{{\rm{r}}})+{\O }_{{\rm{r}}}+\chi {{\O }_{{\rm{r}}}}^{2}}{2{V}_{{\rm{s}}}(\frac{2}{3}{\O }_{{\rm{r}}}-{{\O }_{{\rm{r}}}}^{\frac{1}{3}})}$$

where *χ* is the Flory-Huggins polymer-solvent interaction parameter (PDMS-toluene, 0.092), ${\O }$_r_ is the volumetric fraction of polymer at swelling equilibrium and *V*_s_ is the molar volume of the solvent.

## Electronic supplementary material

Supplementary Information
