# Supplementary material for: Recyclable Polydimethylsiloxane Network Crosslinked by Dynamic Transesterification Reaction
Source: Sci Rep. 2017 Sep 19;7:11833. doi: 10.1038/s41598-017-11485-6 (PMC5605709; doi:10.1038/s41598-017-11485-6)
Supplement: Supplementary file 1 — Supplementary Information [file 41598_2017_11485_MOESM1_ESM.pdf]

Supplementary Information

Recyclable Polydimethylsiloxane Network Crosslinked by Dynamic Transesterification Reaction

Huan Zhang, Chao Cai, Wenxing Liu, Dongdong Li, Jiawei Zhang, Ning Zhao* and Jian Xu*

Contents

Figure S1. FT-IR spectra of pripol 1017, PDMS-diglycidyl ether and the crosslinked PDMS network.

Figure S2. ^{1}H NMR spectra of pripol 1017, PDMS-diglycidyl ether and crosslinked the PDMS network.

Figure S3. Temperature sweeps for the crosslinked PDMS network.

Figure S4. TGA curves for the crosslinked PDMS network.

Figure S5. Strain-recovery ratio as a function of temperature and creep compliance under different stress levels for the crosslinked PDMS network.

Figure S6. Photographic images of the original and the recycled crosslinked PDMS films.

Figure S7. Stress-strain curves of the original and the recycled (non-annealing) crosslinked PDMS samples.

Figure S8. ATR-FT-IR spectra of the original and the recycled crosslinked PDMS network.

Figure S9. Maximal stress and Young modulus of the original and the recycled crosslinked PDMS samples.

Table S1. Swelling properties of the original and the recycled crosslinked PDMS

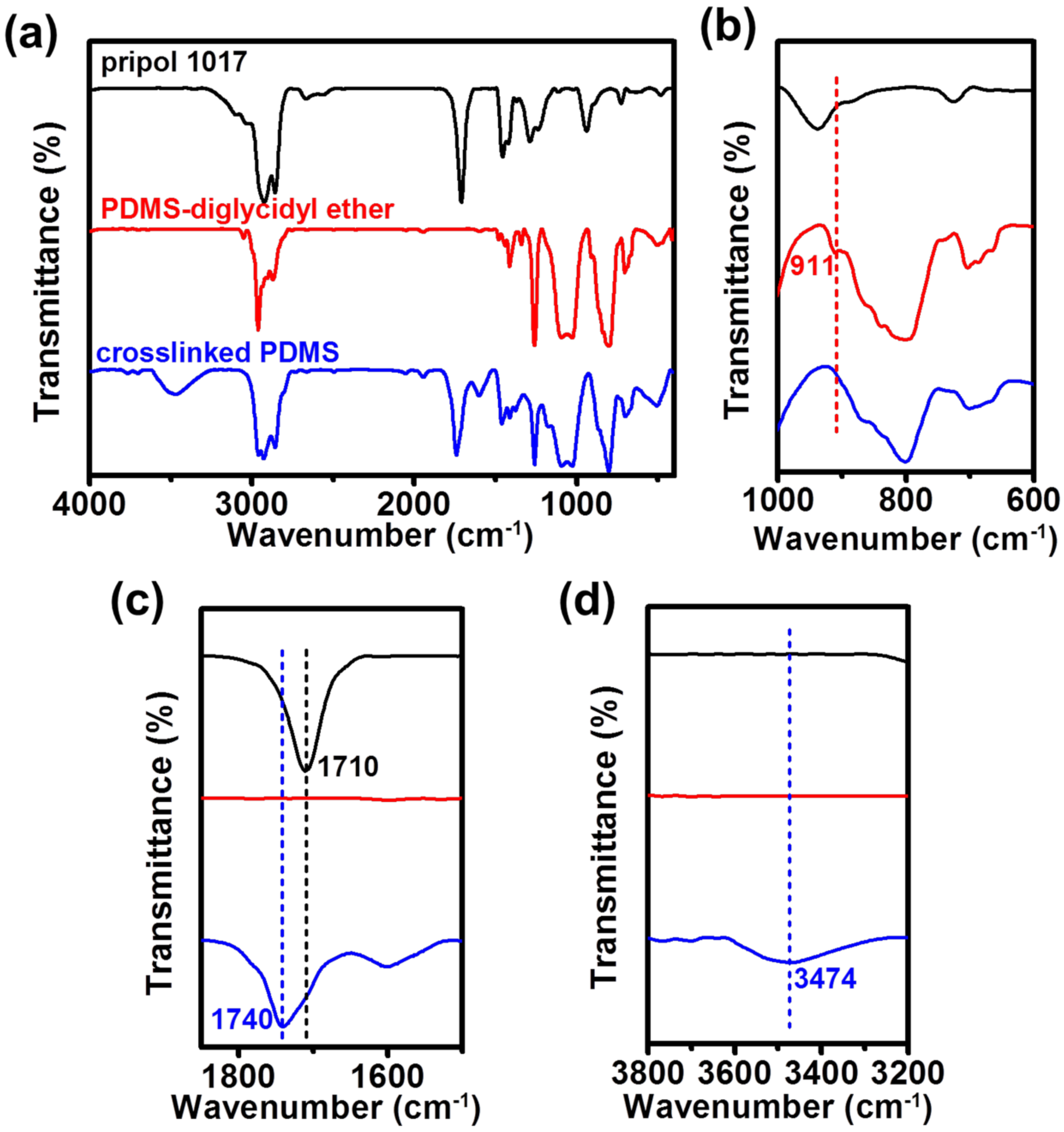

Figure S1 FT-IR spectra of pripol 1017 (black line), PDMS-diglycidyl ether (red line) and the crosslinked PDMS network (blue line) in the range of (a) 400-4000 cm^{-1}, (b) 600-1000 cm^{-1}, (c) 1500-1850 cm^{-1} and (d) 3200-3800 cm^{-1}.

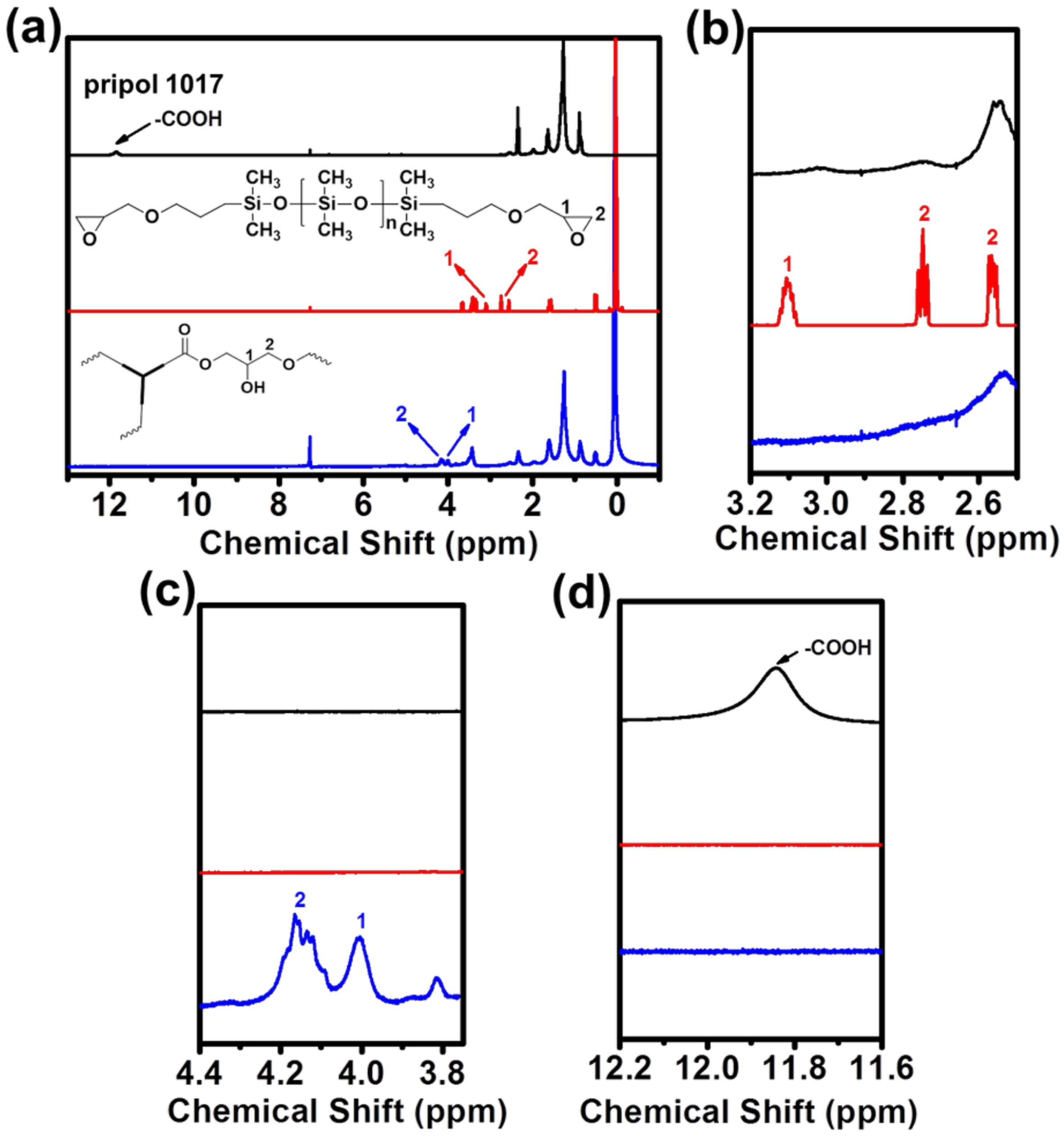

Figure S2 ^{1}H NMR spectra of pripol 1017 (black line), PDMS-diglycidyl ether (red line) and crosslinked the PDMS network (blue line) in the range of (a) 0-13 ppm, (b) 2.5-3.2 ppm, (c) 3.7-4.4 ppm and (d) 11.6-12.2 ppm.

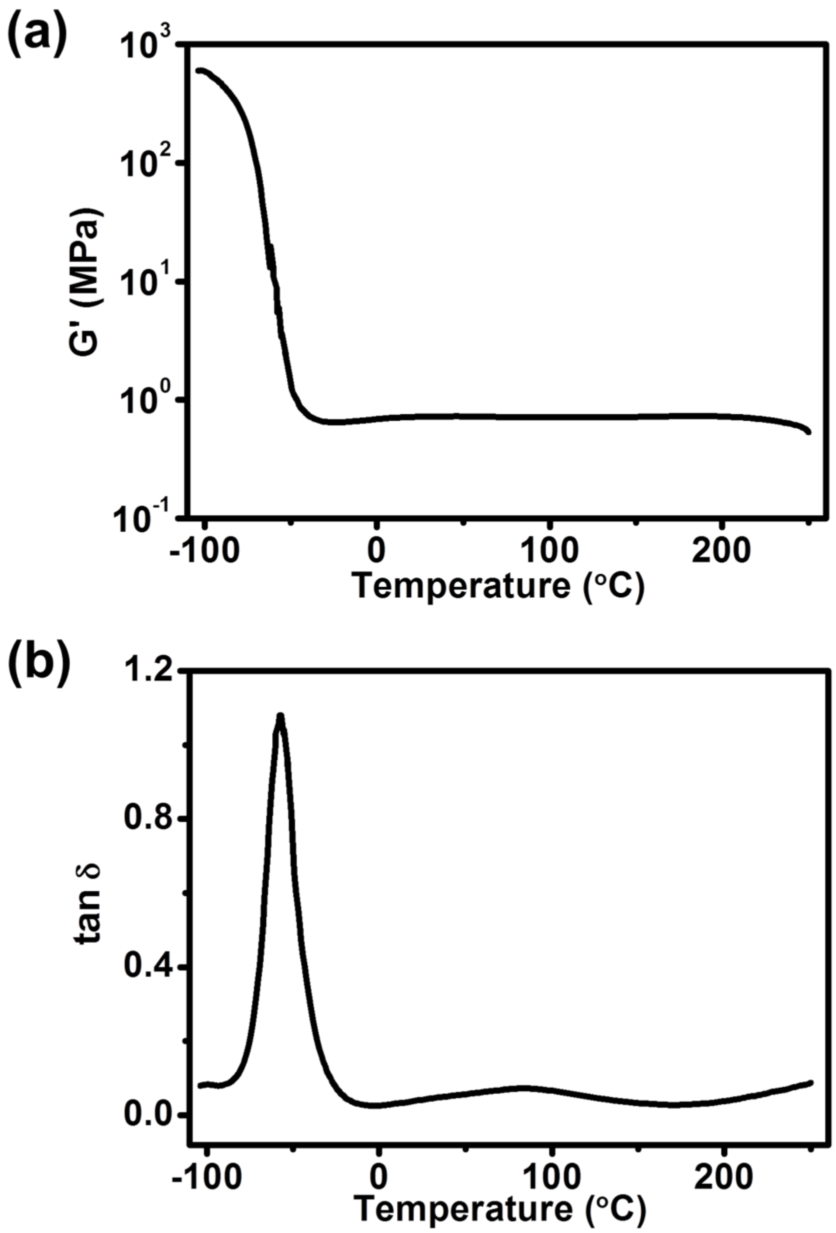

Figure S3 Temperature dependence of (a) storage modulus (G′) and (b) loss factor (tan δ) for the crosslinked PDMS network.

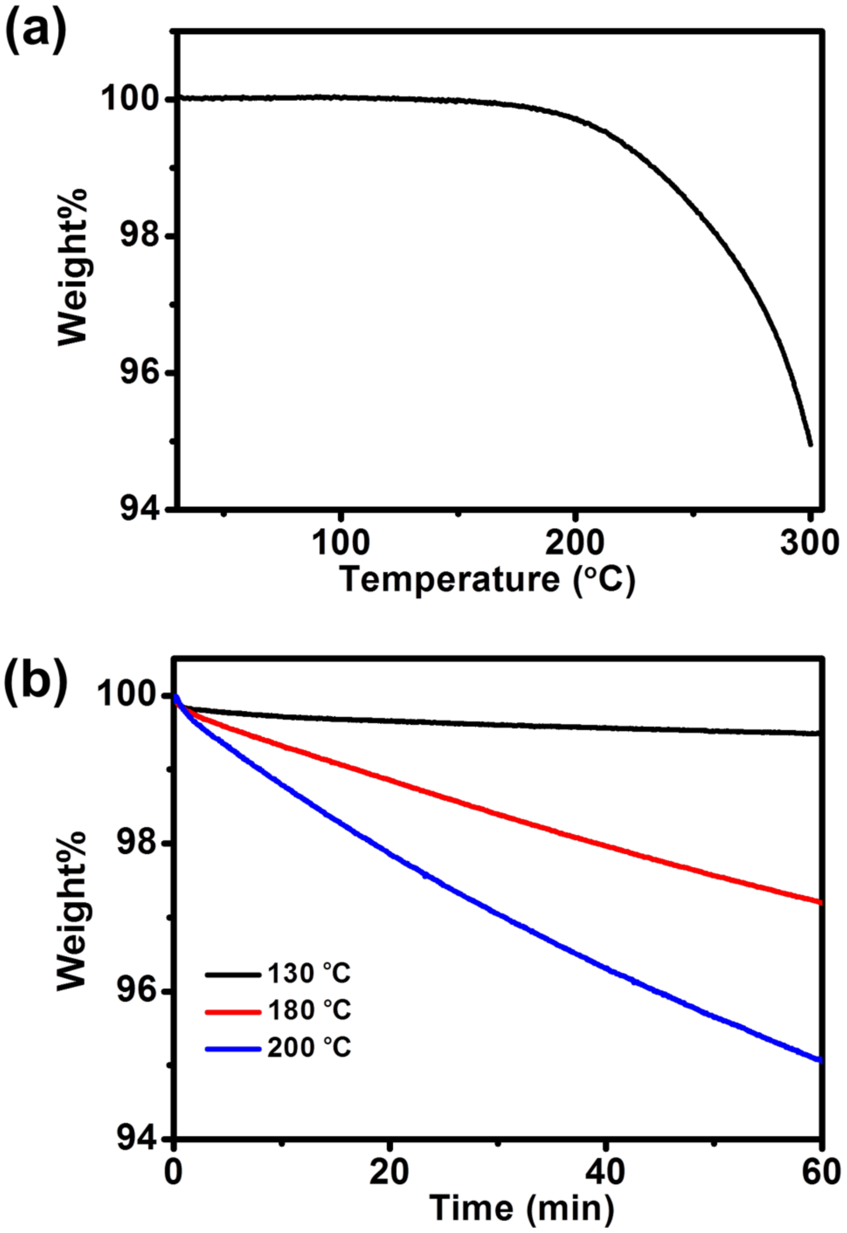

Figure S4 (a) TGA and (b) isothermal TGA at 130, 180 and 200 ℃ for the crosslinked PDMS network.

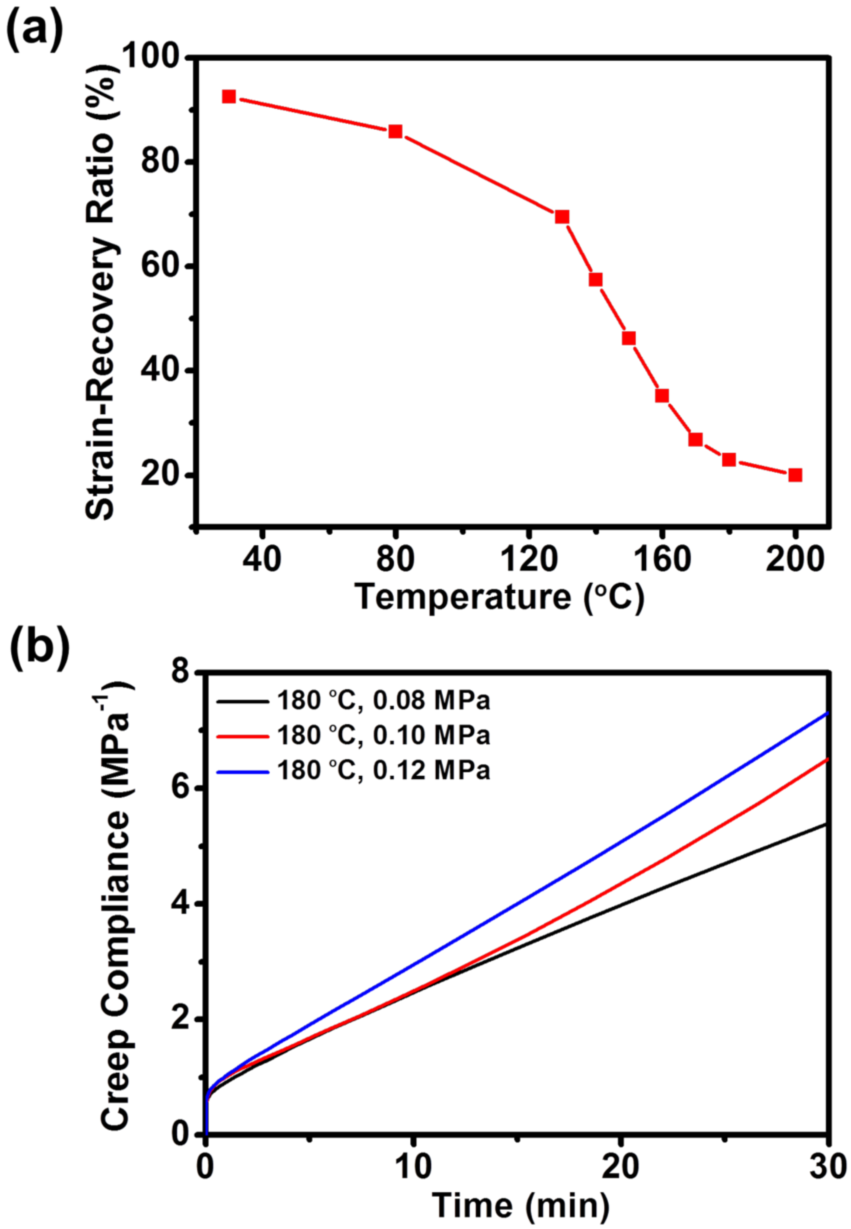

Figure S5 (a) Strain-recovery ratio as a function of temperature and (b) creep compliance under different stress levels at 180 ℃ of the crosslinked PDMS network.

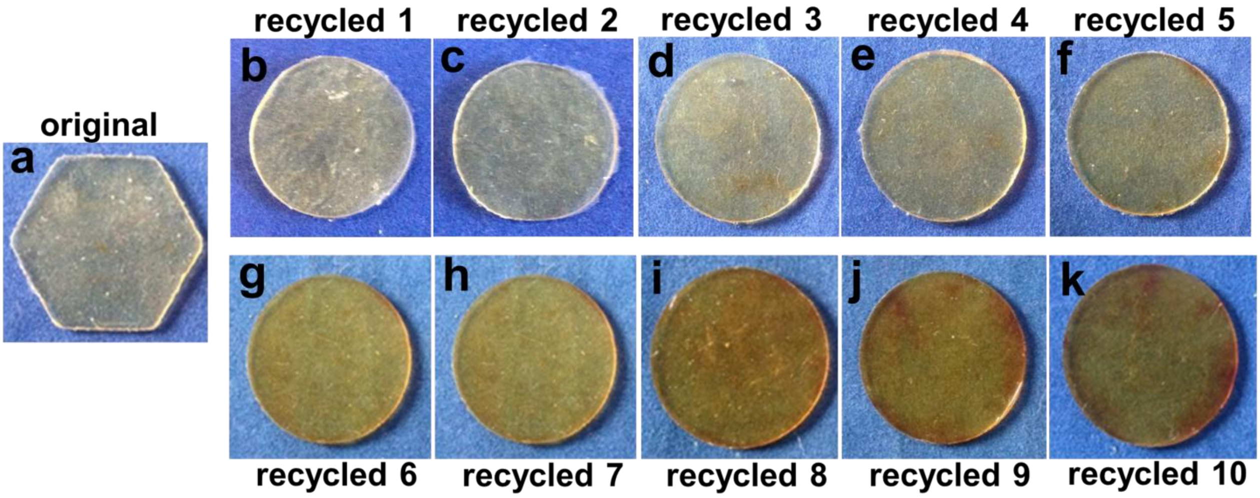

Figure S6 Photographic images of the original and the recycled crosslinked PDMS films.

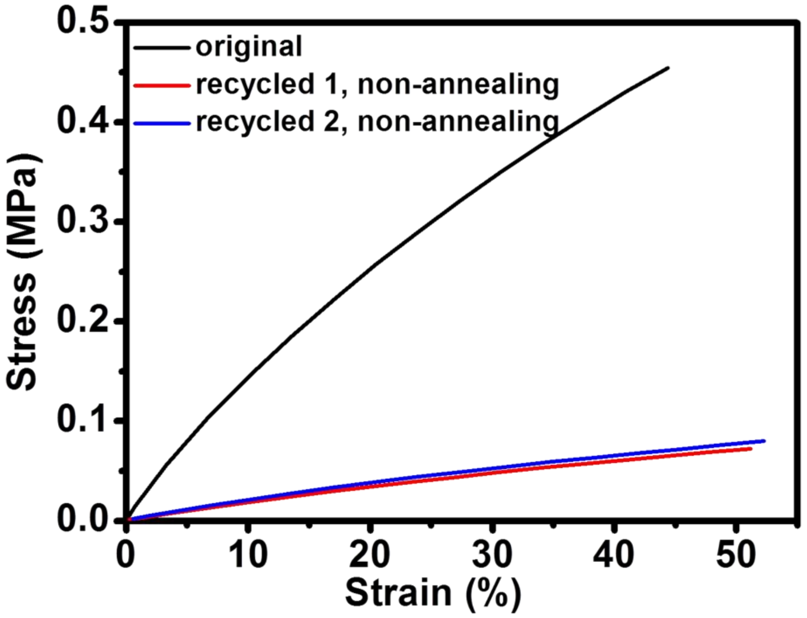

Figure S7 Stress-strain curves of the original and the recycled (non-annealing) crosslinked PDMS samples.

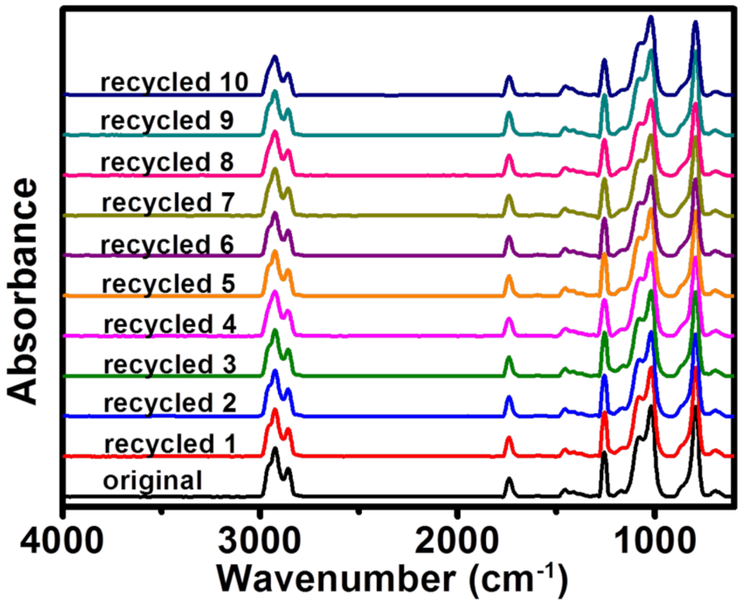

Figure S8 ATR-FT-IR spectra of the original and the recycled crosslinked PDMS network (ten consecutive cycles were proceeded).

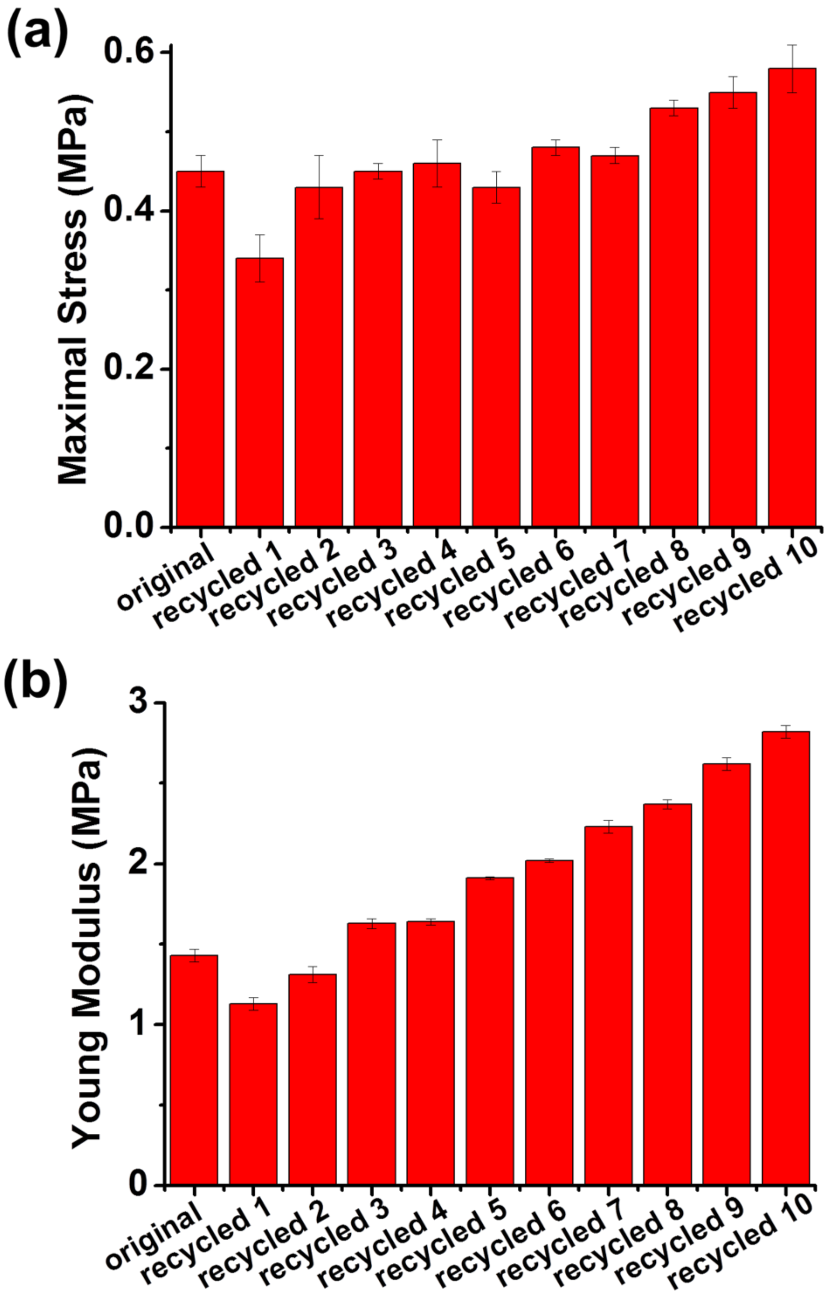

Figure S9 (a) Maximal stress and (b) Young modulus of the original and the recycled crosslinked PDMS samples (ten consecutive recycling cycles were proceeded).

Table S1 Swelling properties of the original and the recycled crosslinked PDMS (ten consecutive recycling cycles were proceeded).

Sample	$m_{initial}$ (mg)	$m_{swollen}$ (mg)	m_{dry} (mg)	*SR* (%)	*GF* (%)	Crosslink Density v (mol/cm^3)
**Original**	26.6	108.4	23.8	355	90	0.0164
**recycled 1**	26.5	109.2	23.2	371	88	0.0143
**recycled 2**	26.6	108.0	23.4	362	88	0.0146
**recycled 3**	26.3	102.2	23.5	335	90	0.0165
**recycled 4**	26.5	103.2	23.6	337	89	0.0160
**recycled 5**	26.6	95.2	23.9	298	90	0.0169
**recycled 6**	26.4	89.7	24.3	269	92	0.0201
**recycled 7**	26.6	86.5	25.1	245	94	0.0248
**recycled 8**	26.2	81.7	24.8	229	95	0.0256
**recycled 9**	26.5	80.5	25.2	219	95	0.0267
**recycled 10**	26.6	79.0	25.4	211	96	0.0279
